# Supplementary figures and images for: Impact of Reactive Sulfur Species on Entamoeba histolytica: Modulating Viability, Motility, and Biofilm Degradation Capacity
Source: Antioxidants (Basel). 2024 Feb 19;13(2):245. doi: 10.3390/antiox13020245 (PMC10886169; doi:10.3390/antiox13020245)

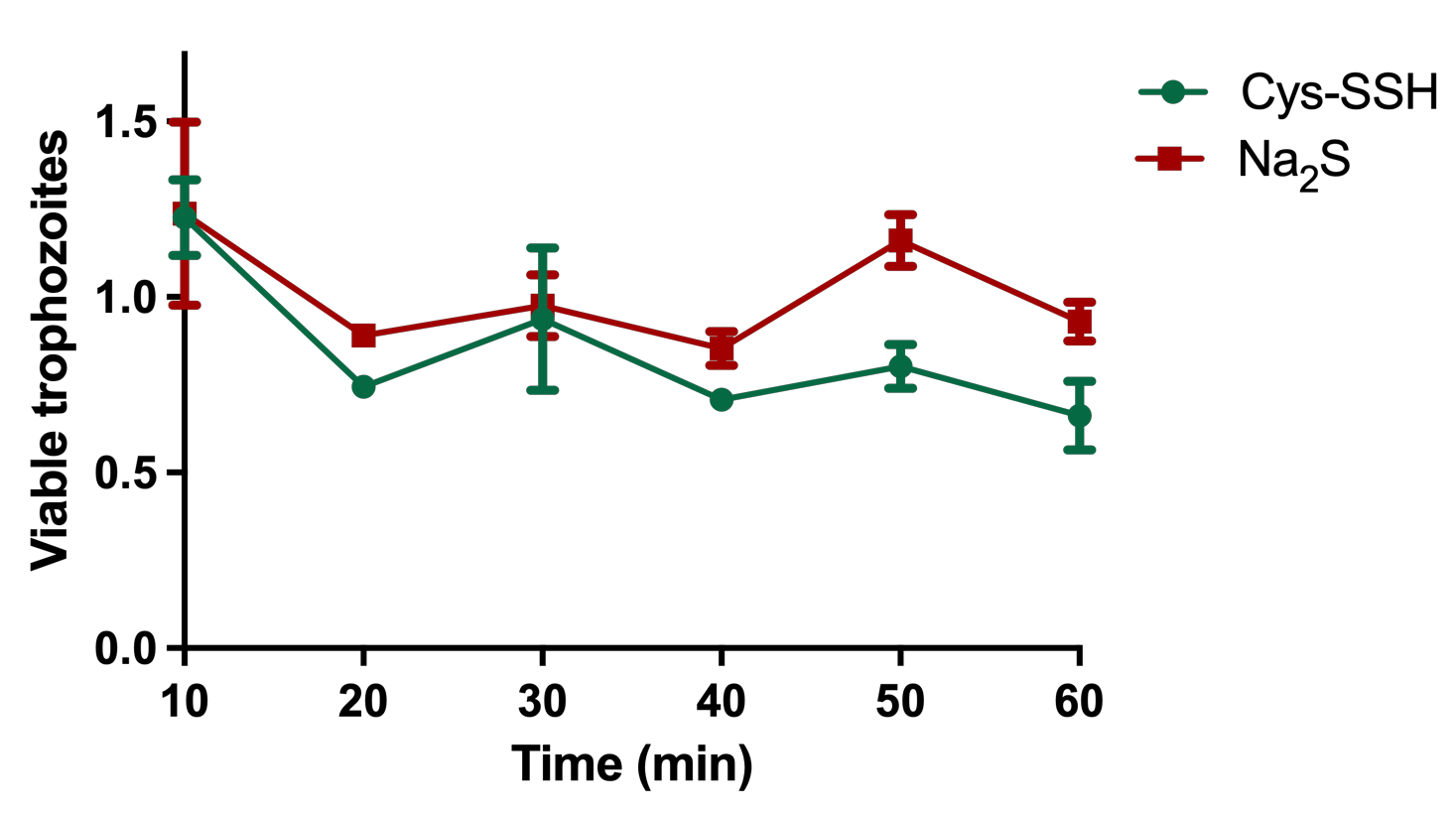

Supplement: Supplementary file 1 [file antioxidants-13-00245-s001.zip › figure S1.tiff]

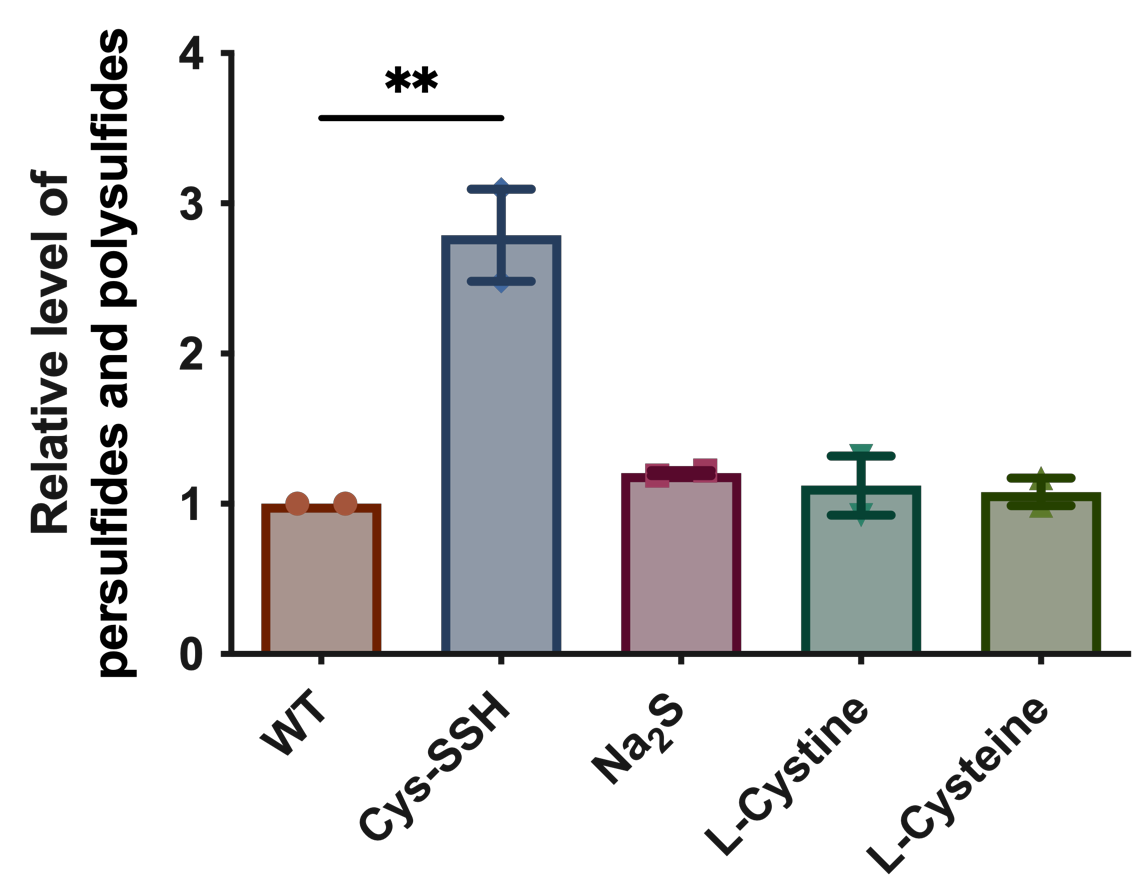

Supplement: Supplementary file 1 [file antioxidants-13-00245-s001.zip › figure S2.tiff]

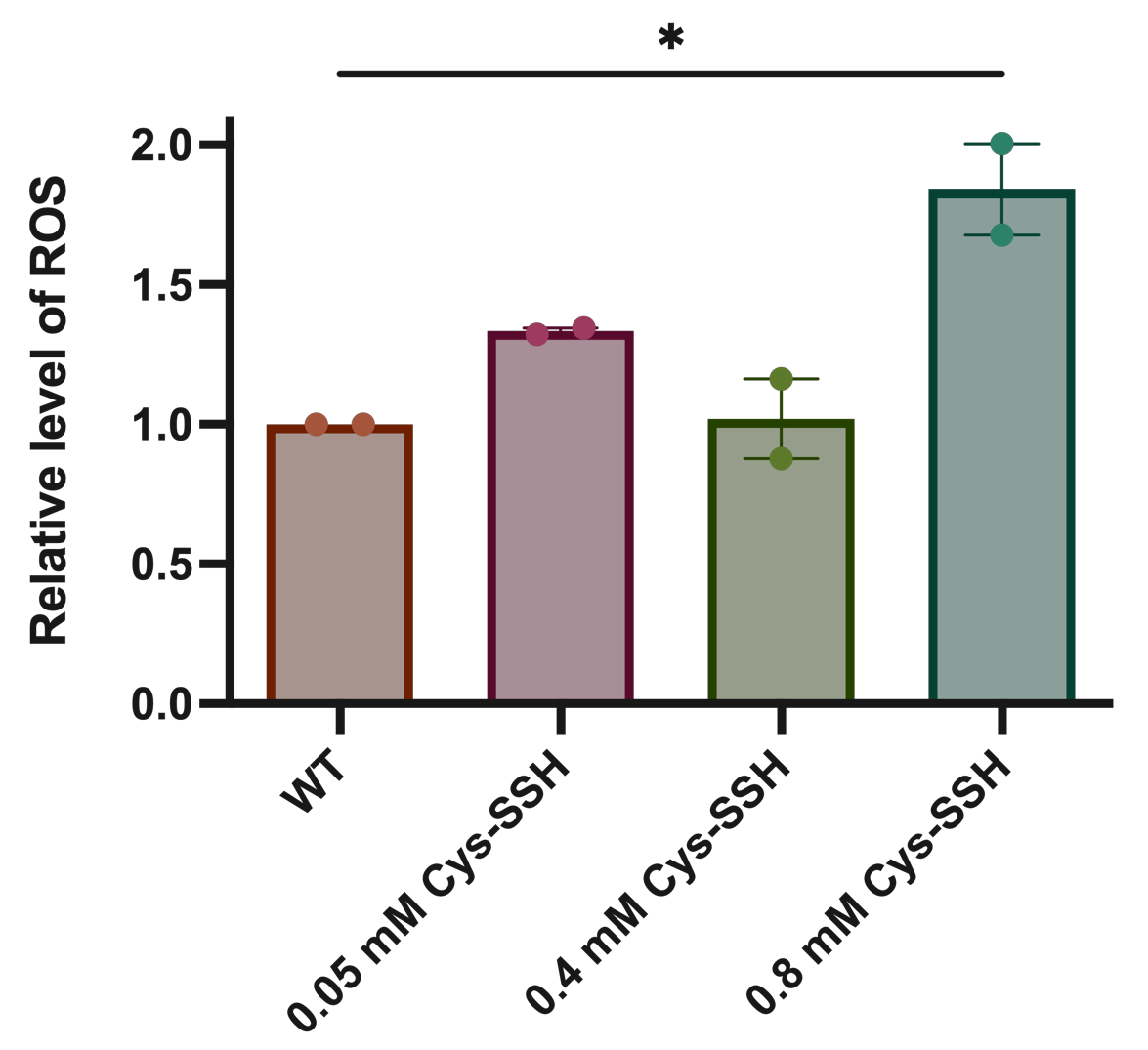

Supplement: Supplementary file 1 [file antioxidants-13-00245-s001.zip › figure S3.tiff]
